# Gualou Guizhi decoction promotes therapeutic angiogenesis via the miR210/HIF/VEGF pathway *in vivo* and *in vitro*

**DOI:** 10.1080/13880209.2023.2204142

**Published:** 2023-05-09

**Authors:** Yuqin Zhang, Yajun Cao, Yan Li, Lijuan Xiao, Wei Xu, Wen Xu, Mei Huang, Xiaoqin Zhang, Yaping Chen, Lihong Nan

**Affiliations:** a Pharmacy College, Fujian University of Traditional Chinese Medicine, Fuzhou, PR China; b Fujian Key Laboratory of Chinese Materia Medica, Fuzhou, China

**Keywords:** Ischemic stroke, neuroprotection, neurological recovery, PKC, eNOS

## Abstract

**Context:**

Gualou Guizhi decoction (GLGZD) is an ancient Chinese classical prescription widely used to treat ischemic stroke. However, the molecular mechanisms of GLGZD promoting angiogenesis are unavailable.

**Objective:**

This study investigates the angiogenesis effect of GLGZD as well as its mechanism.

**Materials and methods:**

Ischemic stroke was established by middle cerebral artery occlusion/reperfusion (MCAO/R) in male Sprague-Dawley (SD) rats. The GLGZD groups received GLGZD (3.6, 7.2 and 14.4 g/kg) orally. Oxygen-glucose deprivation/reoxygenation (OGD/R) model was constructed in HUVECs receiving GLGZD medicated serum (MS). MRI, H&E staining, qRT-PCR, western blot and immunofluorescence methods were employed. miRNA210 inhibitor was employed to confirm the effects of GLGZD on promoting angiogenesis. Dual luciferase assay was used to verify the binding of miRNA210 with HIF mRNA.

**Results:**

GLGZD treatment improved neurological function (by 27%), alleviated neuronal injury (by 76%), reduced infarct volume (by 74%) and increased microvessel density (by fourfold) *in vivo. In vitro* data had also shown that GLGZD caused proliferation of the cells (by 58%), their migration, and eventual formation of tubes (by threefold). Simultaneously, GLGZD enhanced the levels of angiogenesis-related molecules and activated the HIF/VEGF signalling pathway. Surprisingly, the beneficial effects of GLGZD on post-stroke angiogenesis and neurological recovery were weakened by miRNA210 inhibitor, and also abolished the mediation of proangiogenic factors. miRNA210 directly targeted HIF mRNA.

**Discussion and conclusions:**

GLGZD enhances angiogenesis via activation of the miRNA210/HIF/VEGF signalling pathway, suggesting it can be a novel application as an effective angiogenic formula for stroke recovery.

## Introduction

Ischemic stroke, also known as cerebral infarction, is a common type of stroke, and has become the primary cause of death and the dominant cause of disability worldwide, especially in the developed countries (Campbell et al. 2019). Although timely thrombolysis therapy is known to have brought great benefits to patients who have undergone ischemic stroke in the past decades, due to its narrow window of treatment (<4.5 h) and risk of haemorrhagic transformation, its treatment is limited (Kuo et al. 2020). There are no effective drugs in clinic, and the disease always causes severe physical damage and has a poor prognosis, which leads to increased economic and mental burden to patients and their families (Barthels and Das 2020). Thus, several recent studies have focused on developing effective medicine formulations with the quality of neurorestoration to enhance post-stroke tissue repair.

Emerging evidence indicates that establishing new collateral circulation and restoring local blood supply not only provide sufficient oxygen and nutrients for neurons but also offers a good microenvironment for the survival of neurons after cerebral ischemia/reperfusion injury (Uemura et al. 2020). Importantly, angiogenesis was often closely associated with the establishment of collateral circulation, further improving neurofunctional recovery (Arai et al. 2009). Angiogenesis is the effect of new capillaries that sprout or split from pre-existing vessels, and is beneficial for restoration of blood supply providing oxygen, nutrients and trophic factors. In recent years, therapeutic enhancement of angiogenesis has drawn much attention.

Gualou Guizhi decoction (GLGZD) is an ancient Chinese classical prescription from ‘Essentials from the Golden Cabinet’. The formulation of GLGZD is made up of six TCM ingredients, i.e., Trichosanthis Radix [*Trichosanthes kirilowii* Maxim. (Cucurbitaceae) radix; Gualou in Chinese], Paeoniae [*Paeonia lactiflora* Pall. (Ranunculaceae) radix; Baishao in Chinese], Cinnamomum Ramulus [*Cinnamomum cassia* Presl. (Bovidae) ramulus; Guizhi in Chinese], Zingiberis Rhizoma Recens [*Zingiber officinale* Rose. (Zingiberaceae) fresh root; Shengjiang in Chinese], Jujubae Fructus [*Ziziphus jujuba* Mill. (Rhamnaceae) fructus; Dazao in Chinese] and Glycyrrhizae [*Glycyrrhiza uralensis* Fisch. (Fabaceae) radix et rhizoma; Gancao in Chinese], with a weight ratio of 10:3:3:3:2:3. We previously established GLGZD’s quality standards and high-performance liquid chromatography fingerprint (Lin et al. 2015; Xu et al. 2015). GLGZD has been utilized towards muscular spasticity following stroke, epilepsy or spinal cord injury (Zhang and Ai 2005; Sun 2010; Chen et al. 2013; Yang et al. 2013; Zhu 2015; Lin et al. 2018).

Our previous studies reported that GLGZD can improve behavioural function, decrease infarct volume, attenuate BBB permeability and cerebral oedema, and promote neuron survival *in vivo* and *in vitro* (Zhang Y et al. 2014; Lin et al. 2015; Zhang YQ et al. 2015, 2017; Luo et al. 2022). GLGZD exerted significant neuroprotective effects against cerebral ischemia–reperfusion injury, and which may be related with a couple of neuro-protective mechanisms, such as anti-apoptosis, antioxidative damage and anti-excitatory neurotoxicity (Zhang Y et al. 2018, 2021; Nan et al. 2020; Chang et al. 2021). Nevertheless, the molecular mechanism of GLGZD promoting angiogenesis after cerebral ischemia–reperfusion injury has not been clearly elucidated.

Hypoxia-inducible factor-1 (HIF-1) as a transcription factor has recently been demonstrated to promote post-stroke angiogenesis (Yu et al. 2020). HIF-1α is transferred into the nuclei of cells where HIF-1α is bound to HIF-1β to form the complete active HIF-1 complex under hypoxia conditions. Then, the activation of HIF modulates transcription of hundreds of target genes downstream, like vascular endothelial growth factor (VEGF), endothelial nitric oxide synthase (eNOS), etc. These genes are involved in the cell proliferation, migration and tube formation (Rust 2020). Besides, there is now growing evidence to suggest that some miRNAs play a crucial role in angiogenesis. Reports have revealed that miR210 acts as one of the pivotal miRNAs regulating angiogenesis via HIF (Ren et al. 2016; Bavelloni et al. 2017). Therefore, in the present study, we investigated the pharmacological effects of GLGZD on promoting angiogenesis following cerebral ischemia–reperfusion injury and elucidate its potential mechanism involved in HIF/VEGF.

## Materials and methods

### Preparations of GLGZD and its medicated serum

All Chinese medicinal materials were obtained from Bozhou Yonggang Yinpian Factory Co. Ltd (Bozhou, China). GLGZD was prepared according to our previous experiment (Zhang Y et al. 2014).

Following this, GLGZD medicated serum (MS) was extracted according to a published method (Xiang et al. 2019). A total of 20 Sprague-Dawley (SD) rats were randomly divided into control and GLGZD 14.4 g/kg group (*n* = 10), and anesthetized at 1 h after the last administration, and blood samples were taken from the aseptic abdominal aorta by vacuum blood collection tubes. The blood was first placed at room temperature for 2 h, then centrifuged at 3000 rpm for 15 min. After incubating for 30 min in a water bath at 56 °C, the serum was filtered with 0.22 μm microporous membrane and stored at −80 °C after sterilization. GLGZD MS for treating cells was made ready prior to use by means of dissolving GLGZD MS in medium (v/v) with 2.5%, 5% and 10%.

### Animals

Six-week-old-male SD rats (120) (Shanghai Slac Laboratory Animals Co., Ltd., Shanghai, China) were kept in the SPF animal laboratory of the Animal Experimental Center of Fujian University of Traditional Chinese Medicine. Subsequently, the feeding conditions of rats were: temperature 21–23 °C, humidity 55–75% and 12 h day/night cycle. All animal studies were conducted with the animal ethics approval from The Institutional Animal Care and Use Committee at Fujian University of Traditional Chinese Medicine (ethics approval number FJTCM IACUC 2021087).

### Middle cerebral artery occlusion/reperfusion (MCAO/R) model

The MCAO/R operation was conducted according to our previously conducted studies (Zhang Y et al. 2014). In brief, a monofilament nylon suture with a round silicone tip (3800AAA, Guangzhou Jialing Biotechnology Co., Ltd, Guangzhou, China) was carefully inserted to the left middle cerebral artery (MCA) from the ICA until a slight resistance was felt. After 2 h of occlusion, the nylon suture was withdrawn to allow for blood reperfusion. The sham group underwent the same operative procedures except MCAO. During surgery, rectal temperature was monitored and maintained at 37.0 ± 0.5 °C using a temperature-controlling pad.

To confirm the success of the MCAO/R model quickly, 60 min after awakening from anaesthesia, rats were evaluated by the modified neurological severity scores (mNSS) according to Chen et al. (2001). Only rats with a high-grade neurological deficit (8 or greater) were employed. Herein, 96 rats were included to do the MCAO/R operation randomly. A total of 24 rats were excluded due to death or failing to meet the criteria.

### Experimental design and drug administration

#### Experiment 1

Forty-eight rats of MCAO/R model were randomly divided into four groups: (1) MCAO group: rats undergoing MCAO/R and saline treatment; (2) GLGZD-L group: rats undergoing MCAO/R and GLGZD (3.6 g/kg) treatment; (3) GLGZD-M group: rats undergoing MCAO/R and GLGZD (7.2 g/kg) treatment; (4) GLGZD-H group: rats undergoing MCAO/R and GLGZD (14.4 g/kg) treatment. Twelve other unmodelled rats were set as sham group: rats without insertion of the filament. The administration groups received intragastric administration of GLGZD or saline for seven days beginning at one day after MCAO/R. The dose used in this experiment was selected according to our previous study (Zhang YQ et al. 2015).

#### Experiment 2

Twenty-seven rats were randomly assigned into four groups: (1) MCAO group: rats received HBAAV2/9-ZsGReen by lateral ventricle administration before MCAO/R induction and saline treatment; (2) MCAO + 14.4 g/kg GLGZD group: rats received HBAAV2/9-ZsGReen by lateral ventricle administration before MCAO/R induction and GLGZD (14.4 g/kg) treatment; (3) MCAO + 14.4 g/kg GLGZD + miR210 inhibitor group: rats received HBAAV2/9-CMV-rno-miR-210-3p-sponge-ZsGReen by lateral ventricle administration before MCAO/R induction and GLGZD (14.4 g/kg) treatment. Nine other unmodelled rats were set as sham-NC group: rats received HBAAV2/9-ZsGReen by lateral ventricle administration before sham-operation. The rats were received intragastric administration of GLGZD or saline, and the dose used in this experiment was selected according to a preliminary experiment.

### Neurobehavioural evaluation

To determine neurological functions, mNSS and Ashworth tests were performed before surgery (baseline) as well as on days 3, 5 and 7 after MCAO/R. The treated rats were evaluated by an observer who was blinded to the experimental setting and operation.

### Determination of cerebral infarction

Cerebral infarction was measured by magnetic resonance imaging (MRI) in this study. T2WI maps were collected. Hierarchical region splitting (HRS) was used to automatically identify core and penumbra volumes (total lesion = core + penumbra) from T2 relaxation. Data from each modality were summarized per group.

### Histological assessment

All rats were anesthetized with 5% isoflurane, and transcardially perfused with normal saline and 4% paraformaldehyde (PFA). Then, its brain was removed, and cerebral ischemic area was rapidly embedded in paraffin. Paraffin-embedded tissue sections (5 μm thick) were obtained by microtome, and finally stained with HE and Nissl staining. Histopathological changes were observed under a light microscope.

### Microvascular distribution evaluation

All rats were anesthetized with 5% isoflurane, and transcardially perfused with normal saline, 4% PFA and gelatin-ink solution. Then, its brain was removed, and cerebral ischemic area of the brains was rapidly embedded in paraffin; paraffin-embedded tissue sections (100 μm thick) were obtained by microtome and stained by eosin. The microvascular sections were observed through optical microscope (E100, Nikon, Tokyo, Japan), followed by a quantitative analysis of the proportion of microvascular staining area from five random views on each section.

### Enzyme-linked immunosorbent assay (ELISA)

The brains of all the rats were removed under deep anaesthesia, and ischemic brain tissue was collected and ground with normal saline. The supernatant was obtained by centrifuging at 3500 rpm, and then detecting the content of NO at 550 nm (Infinite M200 Pro; Tecan, Männedorf, Switzerland) according to the manufacture’s protocol.

### Immunohistochemistry staining

Paraffin-embedded tissue sections (5 μm thick) that were obtained by microtome were subsequently dewaxed and rehydrated. Next, antigen retrieval with citrate buffer (50×, BOSTER Biological Technology Co. Ltd., Wuhan, China) and the blocking was done with nonspecial binding sites with goat serum. Then, the sections were incubated with primary antibody against CD34 (1:200, BOSTER Biological Technology Co. Ltd., Wuhan, China) and horseradish peroxidase-linked secondary antibody (1:500, Thermo Fisher Scientific, Waltham, MA). Finally, the samples were incubated with diaminobenzidine (DAB) for chromogenic reaction and then counterstained with haematoxylin. The results of the experiment were based on the five areas of positivity observed, and the proportion of positive staining area was calculated by Image J IHC Profiler software.

### Immunofluorescence staining

As with immunohistochemistry staining, sections of HUVECs were incubated in a dark box with HIF primary antibody (ab197493, Abcam, Danvers, MA), and appropriate fluorescence-conjugated secondary antibody (Beijing Zhongshan Jinqiao Biotechnology Co. Ltd., Beijing, China). DAPI reagent was used to stain the cell nucleus. All of the images were obtained using fluorescence camera microscopy (Leica, Wetzlar, Germany). The results were based on the five areas of positivity observed, and the proportion of positive staining area was calculated by Image J IHC Profiler software.

### Cell culture and oxygen-glucose deprivation/reoxygenation (OGD/R)

Human umbilical vein endothelial cell (HUVEC) was procured from Procell Life Science & Technology Co. Ltd. (Wuhan, China) and cultured in DMEM medium (Gibco, Carlsbad, CA) supplemented with 10% FBS (Gibco, Carlsbad, CA) as needed. HUVEC within 2–5 generations of subculture were inoculated in 96-well or six-well culture plates. Following this, cell confluence reached 50–60% prior to treatment. After 12 h of serum-starving, HUVECs were cultured in a glucose-free DMEM medium and exposed to oxygen-glucose deprivation (OGD) [environment (v/v) 94% N_2_, 5% CO_2_ and 1% O_2_] for 4 h. Following OGD, HUVECs were cultured in glucose-containing medium under standard conditions of 95% O_2_ and 5% CO_2_ for 8 h.

### Experimental design and GLGZD MS treatment

For cell viability assay, HUVECs were partitioned into the following groups: control, OGD/R, OGD/R + 2.5% GLGZD MS, OGD/R + 5% GLGZD MS and OGD/R + 10% GLGZD MS.

For other experiments, HUVECs were partitioned into the following groups: control ± micrOFF inhibitor negative control (NC), OGD/R ± micrOFF inhibitor NC, OGD/R + 10% GLGZD MS and OGD/R + 10% GLGZD MS + micrOFF miR210 inhibitor.

Working GLGZD MS dilutions (GLGZD MS in medium (v/v) with 2.5%, 5% and 10%, which was determined comprehensively considering use in the preliminary experiment) in serum-free medium were prepared and used to pretreat cultures for 24 h prior to exposure to OGD/R. These conditions were maintained throughout the duration of the experiment. Control cells were incubated with an equal amount of medium.

### Cell viability detection

HUVECs were cultured in 96-well plates at a density of 1 × 10^5^/mL to test cell viability. After MS treatment and OGD/R, cell viability was evaluated using CCK8 reagent according to the manufacturer’s instructions. The absorbance was recorded at 570 nm using a microplate reader. Cell viability was obtained by calculating the ratio of absorbance between the treatment groups and the control group.

### Scratch assay

HUVECs were cultured in 24-well plates at a density of 2.5 × 10^5^/mL, and a single scratch in every group was carefully made with a sterile 10 μL pipette tip. After MS treatment and OGD/R, images of the scratches were captured with an Olympus IX71 inverted microscope (Tokyo, Japan). The width of the scratch was analysed.

### Tube formation assay

HUVECs were cultured in 24-well plates which were polymerized by Matrigel (BD Biosciences, Bedford, MA) at a density of 2.5 × 10^5^/mL. After MS treatment and OGD/R, capillary tubes were captured and quantified via measuring the total lengths of the completed tubule structure.

### Western blotting

Ischemic brain tissue (cell protein) extract was assayed with a BCA kit according to the manufacturer’s protocols towards obtaining accurate concentration of proteins and then added with 1/4 volume of protein buffer and denatured by heating. Aliquots of the samples (40 μg) were analysed through SDS-PAGE gel. Following this, the gel was transferred and then blocked and incubated with different corresponding primary antibodies against HIF-1α (ab197493, Abcam, Danvers, MA), VEGF (A12303, ABclonal, Wuhan, China), VEGFR2 (YT5845, Immunoway, Plano, TX), PLCγ1 (AF8390, Affinity, San Francisco, CA), PKC (YT3752, Immunoway, Plano, TX), eNOS (CPA9156, Bejing, China) overnight. Subsequently, the target membranes were incubated with a secondary antibody. Proteins were detected using the ECL kit and the results were analysed using Image Lab analysis software (Bio-Rad Laboratories, Inc., Hercules, CA).

### Quantitative real-time PCR

The Trizol method (Trizol reagent) (Invitrogen, Barcelona, Spain) was employed to extract the total RNA from quick-frozen tissues or cells and aliquots of total RNA (1 μg) was reverse-transcribed with the PrimeScript^®^ RT reagent kit (Takara Bio, Inc., Otsu, Japan). Subsequently, quantitative real-time PCR was carried out with the 7900 real-time PCR system (Applied Biosystems, Inc., Foster City, CA) according to the manufacturer’s instructions. The primer sequences were miR210 F: 5′-CTGTGCGTGTGACAGCGGCT-3′, R: 5′-AGTGCAGGGTCCGAGGTTT-3′; U6 F: 5′-GCGCGTCGTGAAGCGTTC-3′, R: 5′-AACGCTTCACGAATTTGCGT-3′. Relative mRNA expression levels of target genes were calculated according to threshold cycle (Ct) value based on 2^−ΔΔC^_t_ formula using U6 as internal control for sample normalization.

### Dual-luciferase reporter assay

HUVECs were cultured in six-well plates at a density of 2.5 × 10^5^/mL, and were co-transfected with the recombinant plasmid WT-HIF together with miRNA210 mimics or mimics negative control (NC) (synthesized from Genepharma, Shanghai, China), MUT-HIF together with miRNA210 mimics or miR-NC, by Lipofectamine™ 2000 Liposome method. Then, 6 h thereafter, the co-transfected medium was replaced with normal medium, then continually incubated for another 24 h, and cells were harvested in the reporter lysis buffer. The luciferase activity was measured by the Dual-Luciferase Reporter Assay kit (Beyotime, Jiangsu, China).

### Statistical analysis

All results of the experiment were shown as means ± SD. SPSS software 21.0 (SPSS Inc., Chicago, IL) was used to analyse the data and one-way ANOVA followed by a *post hoc* LSD, and a Games-Howell test was used to analyse the differences. The difference was designated as statistically significant when *p* value was <0.05.

## Results

### GLGZD provides neuroprotective effects against cerebral ischemic injury in rats

**Figure 1.**
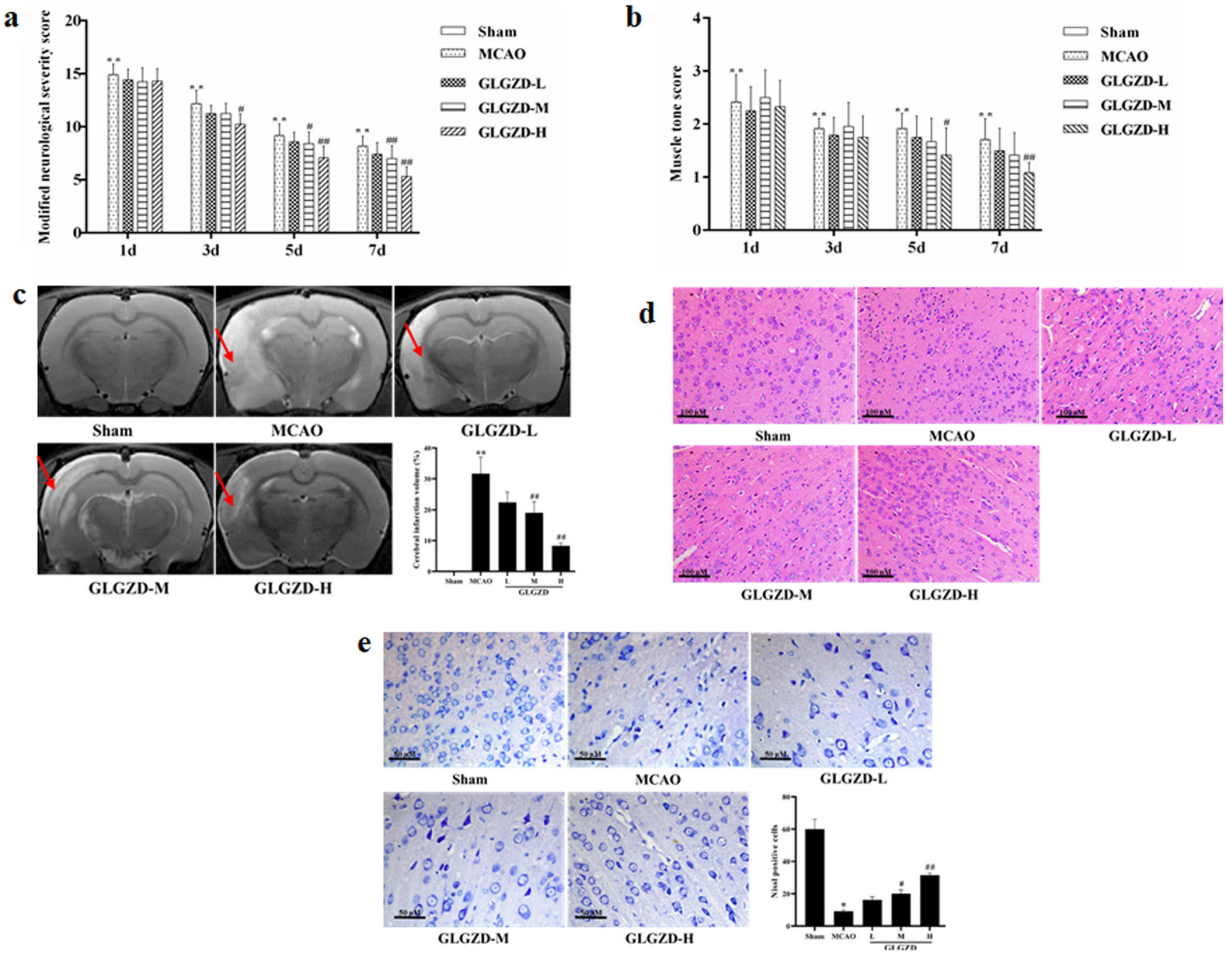
GLGZD provides neuroprotective effects against cerebral ischemic injury in rats. (a, b) Neurologic deficit evaluation by mNSS and Ashworth test in rats for each group. (c) MRI and infarct volume of each group, red arrows indicate infarction area. (d, e) HE and Nissl stained images for pathological assessment in rats for each group (×200, scale bar = 100 μm). Data are presented as mean ± SD. **p < 0.01 vs. sham, ^#^*p* < 0.05, ^##^*p* < 0.01 vs. MCAO.

The impact of GLGZD on cerebral ischemic injury in rats, neurologic deficit, area of cerebral infarction, histological and morphological assessments were evaluated. Results of neurologic deficit evaluation by mNSS and Ashworth test revealed that the mNSS points and the percentage of right turns of the GLGZD group decreased compared to the MCAO group after GLGZD administration at days 3, 5 and 7, especially GLGZD-M and GLGZD-H group (*p* < 0.05 or *p* < 0.01) (Figure 1(a,b)). Also, as shown in Figure 1(c), rats in the model group displayed obvious infarct (*p* < 0.01), in contrast, cerebral infarction in the GLGZD treatment group was significantly reduced (*p* < 0.05 or *p* < 0.01). Moreover, as can be seen from Figure 1(d,e), MCAO resulted in obvious karyopyknosis, neuronal damage or neuronal loss, which was remarkably converted after GLGZD treatment. These obtained results indicating the GLGZD had favourable neuroprotective effects.

### GLGZD enhances angiogenesis after cerebral ischemic injury in rats

**Figure 2.**
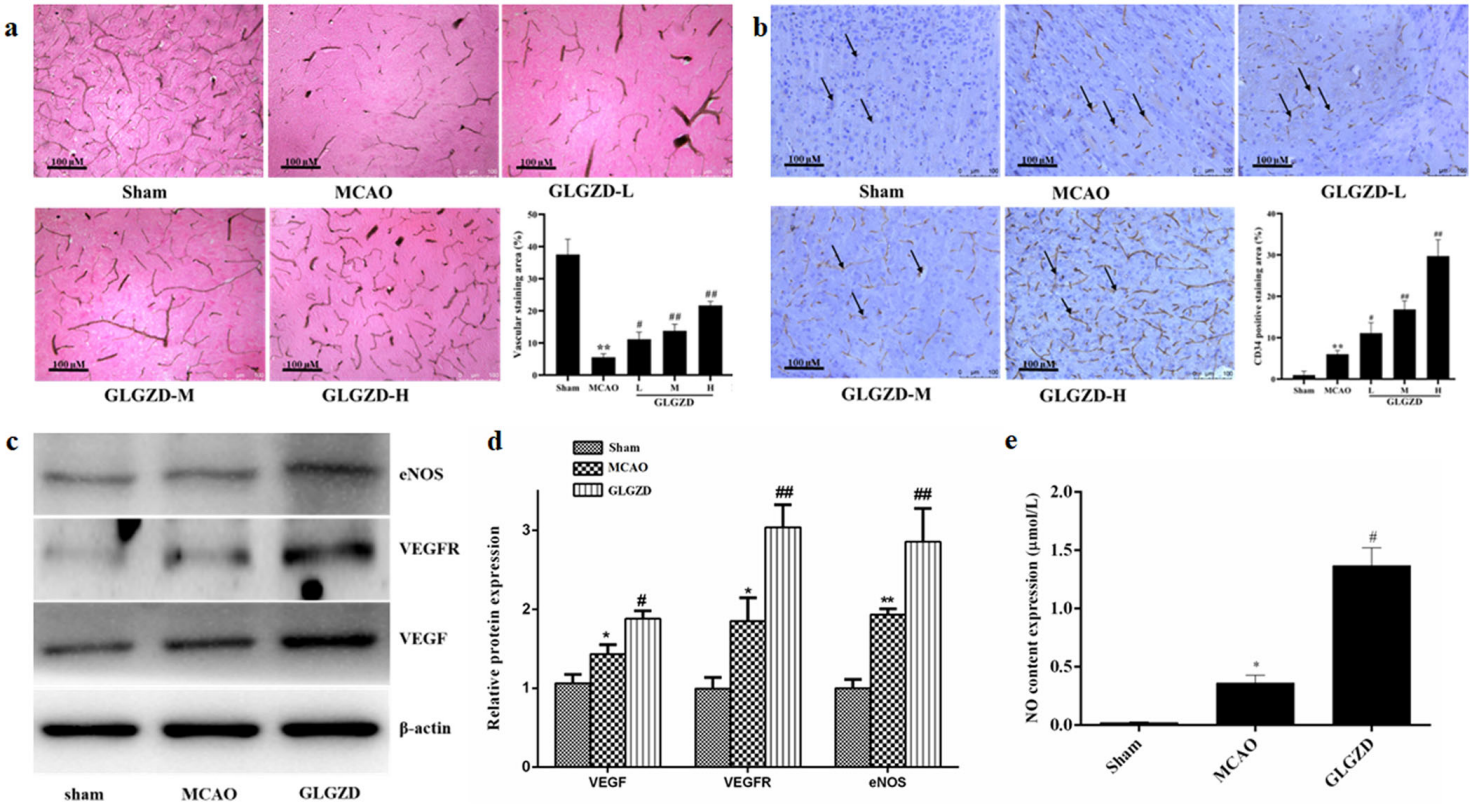
GLGZD enhances angiogenesis after cerebral ischemic injury in rats. (a) Representative photographs and quantification analysis of microvascular staining in different groups of rats (×200, scale bar = 100 μm). (b) Representative photographs and quantification analysis of immunohistochemistry staining of CD34 (indicated by black arrows) in different groups of rats (×200, scale bar = 100 μm). (c, d) Western blotting results for relative protein expression of VEGF, VEGFR2 and eNOS in different groups of rats. (e) The content of NO in different groups of rats. Data are presented as mean ± SD. *p < 0.05, **p < 0.01 vs. sham, ^#^*p* < 0.05, ^##^*p* < 0.01 vs. MCAO.

Enhanced angiogenesis is considered an important role in improving brain blood supply after cerebral ischemia. Therefore, the impact of GLGZD on the blood vessels distribution and vascular density was examined around the ischemic lesion. As shown in Figure 2(a,b), the blood vessels distribution and the number of microvessels with staining of CD34 in the peri-infarct cortex were obviously increased by GLGZD treatment compared with the MCAO group (*p* < 0.05 or *p* < 0.01). Additionally, NO, eNOS, VEGF and VEGFR were detected in brain that were dominant proangiogenic factors. In comparison with the sham group, the protein levels of eNOS, VEGF and VEGFR were significantly increased in the MCAO group, and the levels were further up-regulated by GLGZD treatment (*p* < 0.05 or *p* < 0.01, Figure 2(c,d)). NO level also demonstrated the synchronous trend with the expressed proteins (*p* < 0.05 or *p* < 0.01, Figure 2(e)). In conclusion, we speculated that GLGZD promoted angiogenesis after cerebral ischemic injury, which may provide the basis for tissue regeneration.

### GLGZD enhances endothelial cell viability, migration and tube formation after hypoxia *in vitro*

**Figure 3.**
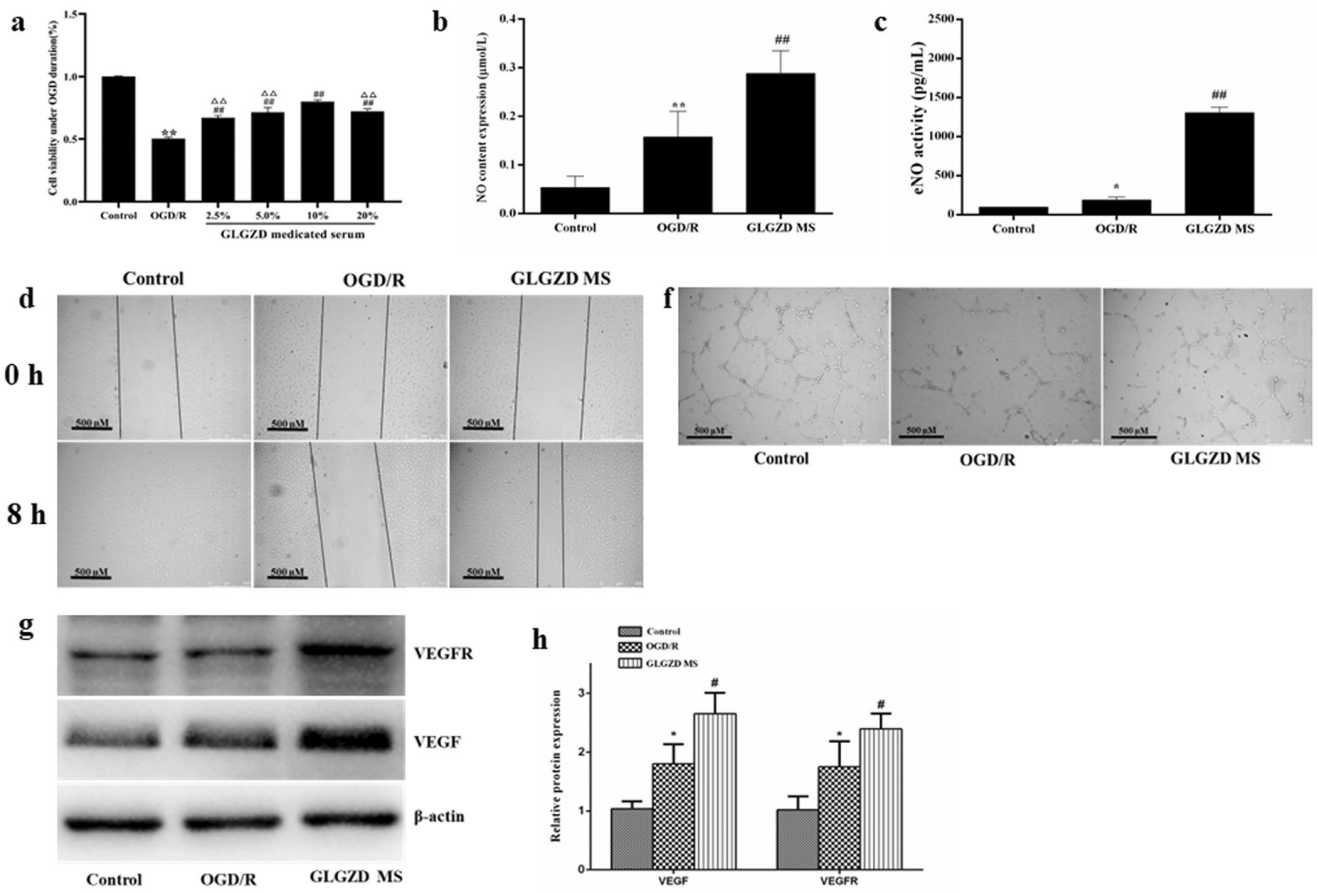
GLGZD enhances endothelial cell viability, migration and tube formation after hypoxia *in vitro*. (a) The results of CCK8 cell viability assay in HUVECs after OGD/R for each group. (b) The result of scratch assay in HUVECs after OGD/R for each group. (c) Representative photographs of tube formation in HUVECs after OGD/R for each group (scale bar = 500 μm). (d) The content of NO and eNOS in HUVECs after OGD/R for each group. (e, f) Western blotting results for relative protein expression of VEGF and VEGFR2 in HUVECs after OGD/R for each group. Data are presented as mean ± SD. *p < 0.05, **p < 0.01 vs. control, ^#^*p* < 0.05, ^##^*p* < 0.01 vs. OGD/R, ^ΔΔ^*p* < 0.01 vs. 10% GLGZD MS.

The corresponding *in vitro* biological functions of GLGZD on promoting the cell viability and tube formation of HUVECs after OGD/R were investigated. CCK8 cell viability assay showed that HUVECs were significantly damaged after OGD/R, and was ameliorated following the GLGZD MS treatment in a dose-dependent manner (Figure 3(a), *p* < 0.01), especially GLGZD MS in medium (v/v) with 10%. Thus, GLGZD MS in medium (v/v) with 10% was selected to explore the effect of GLGZD on endothelial cell migration and tube formation after hypoxia in the subsequent experiments.

The result of scratch assay showed that the GLGZD MS treatment notably promoted proliferation and migration when compared with the OGD/R group after 8 h of co-culture (Figure 3(d), *p* < 0.01). Meanwhile, tube formation of HUVECs was also exhibited, as shown in Figure 3(f), GLGZD MS treatment obviously mediated facilitating effect on tube formation of HUVECs in comparison with the OGD/R group (*p* < 0.01). Furthermore, the pro-angiogenic effects were elucidated to be linked to the upregulations of NO, eNOS, VEGF and VEGFR in a dose-dependent manner (Figure 3(b,c,g,h), *p* < 0.01).

### GLGZD activates miR210/HIF1α/PKC signalling pathway in the angiogenesis process

**Figure 4.**
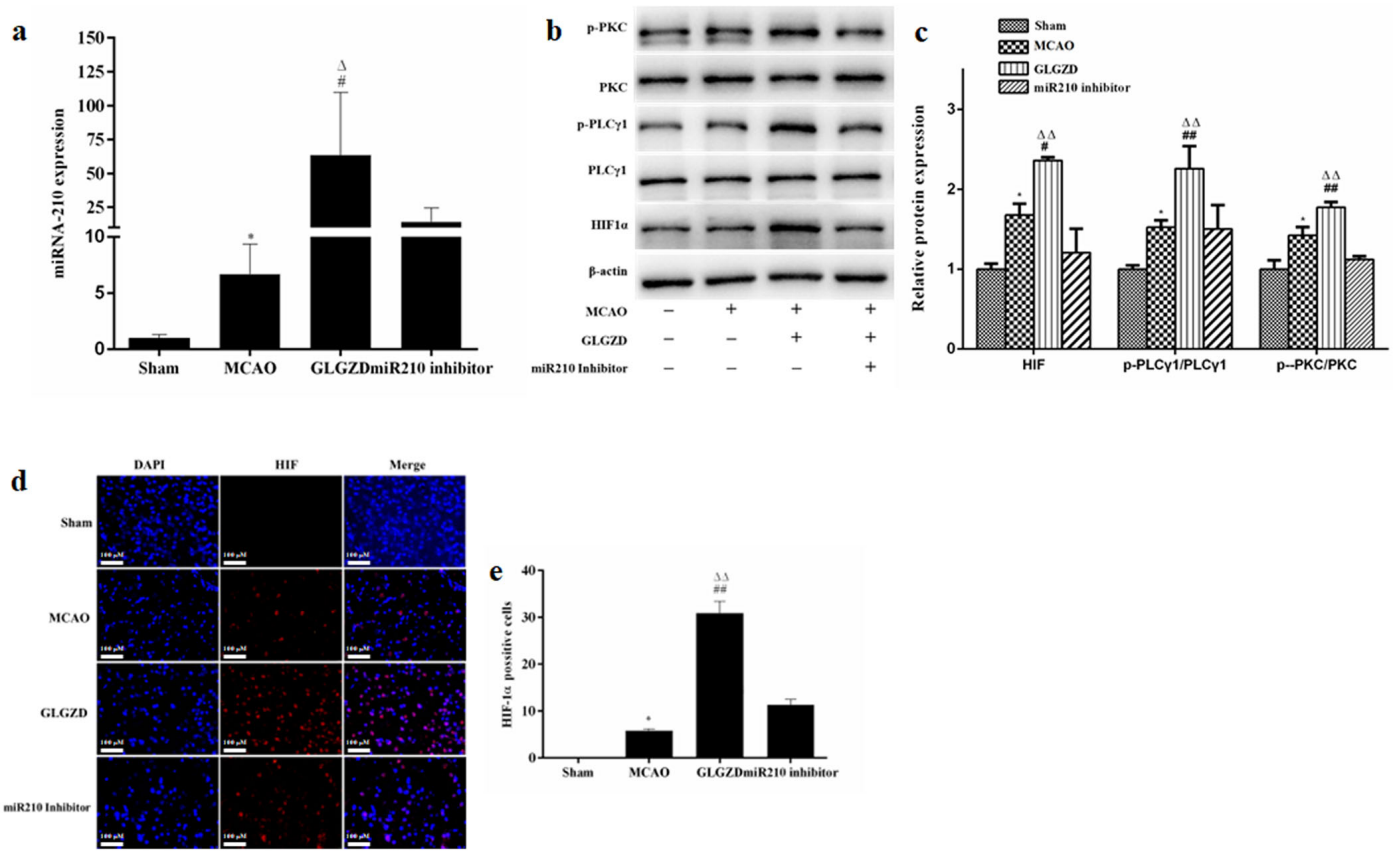
GLGZD activates the miR210/HIF1α/VEGF signalling pathway after cerebral ischemic injury in rats. (a) The expression of miR210 mRNA was determined by quantitative RT-PCR in rats for each group. (b) Western blotting results for relative protein expression of the HIF1α/PKC signalling pathway in rats for each group. (c) Images of HIF immunofluorescence staining of the ischemic area of the cortex in different groups (×200, scale bar = 100 μm). Data are presented as mean ± SD. *p < 0.05 vs. Sham, ^#^*p* < 0.05, ^##^*p* < 0.01 vs. MCAO, ^Δ^*p* < 0.05, ^ΔΔ^*p* < 0.01 vs. miR210 inhibitor.

Based on these results, we selected 14.4 g/kg of GLGZD for the subsequent experiments to explore the potential underlying mechanisms involved in the proangiogenic outcomes induced by GLGZD. Real-time PCR analysis showed that there was a marked up-regulation of miR210 in the GLGZD group compared to the MCAO group (*p* < 0.01) (Figure 4(a)). Western blotting results showed that GLGZD increased HIF1α protein level compared to the MCAO group (*p* < 0.01) (Figure 4(b,c)). In line with the result of Western blot, immunofluorescent staining results exhibited that the immunoreactivity of HIF1α was robust in the GLGZD group (*p* < 0.01) (Figure 4(d,e)). In addition, the phosphorylated levels of PKC and PLCγ1 (p-PKC and p-PLCγ1) were significantly enhanced after GLGZD treatment compared to the MCAO group (*p* < 0.01) (Figure 4(b,c)). However, when miR210 was knocked down (HBAAV2/9-CMV-rno-miR-210-3p-sponge-ZsGReen was administered by lateral ventricle), the HIF1α/PKC signal activation effect of GLGZD was blocked (Figure 4(b,c)).

**Figure 5.**
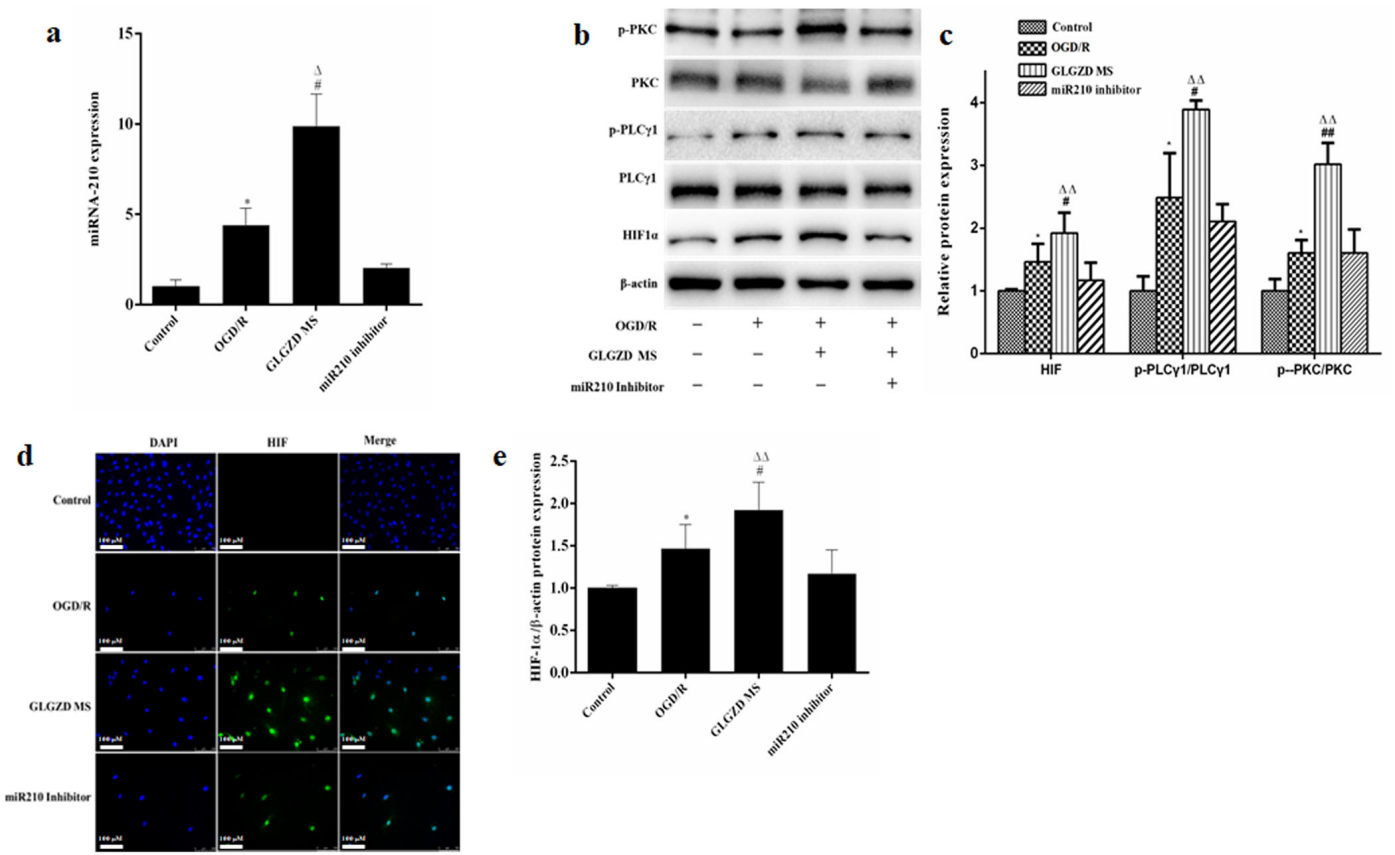
GLGZD activates the miR210/HIF1α/VEGF signalling pathway in HUVECs after OGD/R. (a) The expression of miR210 mRNA was determined by quantitative RT-PCR in HUVECs for each group. (b) Western blotting results for relative protein expression of HIF1α/PKC signalling pathway in HUVECs for each group. (c) Images of HIF immunofluorescence staining of HUVECs in different groups (×200, scale bar = 100 μm). Data are presented as mean ± SD. *p < 0.05 vs. control, ^#^*p* < 0.05, ^##^*p* < 0.01 vs. OGD/R, ^Δ^*p* < 0.05, ^ΔΔ^*p* < 0.01 vs. miR210 inhibitor.

Furthermore, we examined the effect of GLGZD on the miR210/HIF1α/PKC signalling pathway of OGD/R-treated HUVECs. Notably, GLGZD increased the levels of miR210, HIF1α p-PKC and p-PLCγ1, and promoted HIF nuclear transcription compared with the OGD/R group (Figure 5). The results were consistent with the *in vivo* results.

### miR210 inhibitor reverses the GLGZD-mediated promotion of angiogenesis on HUVECs

**Figure 6.**
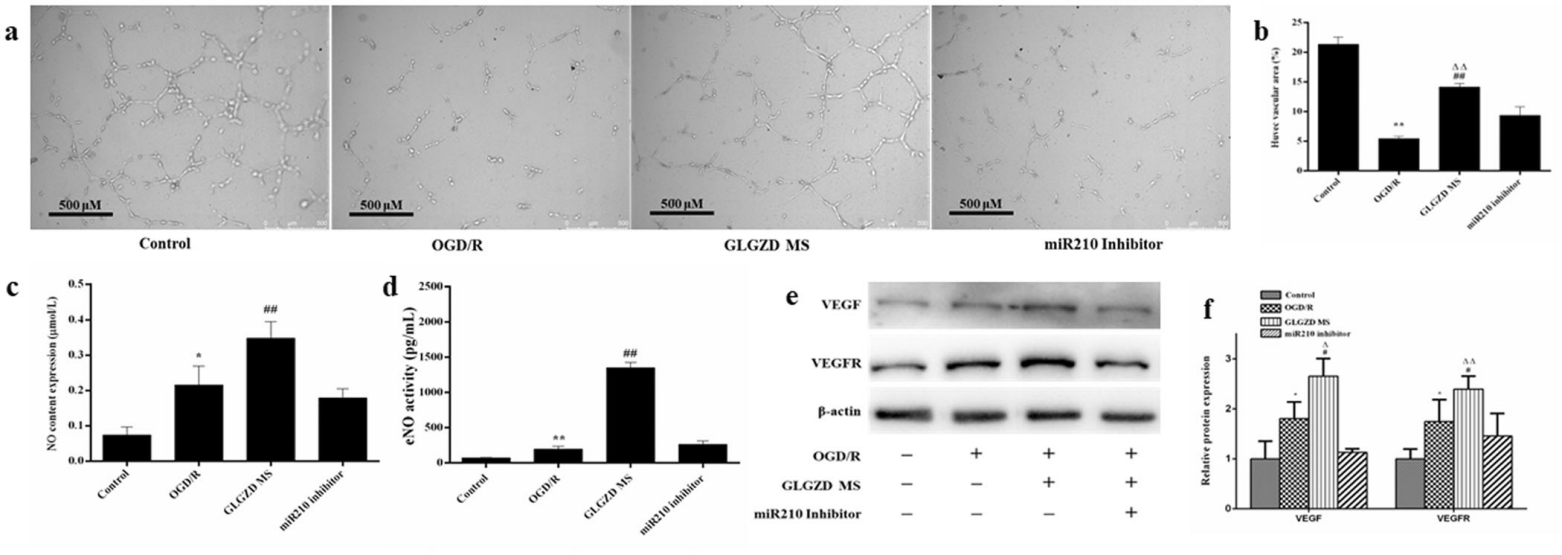
miR210 inhibitor reverses the GLGZD-mediated promotion of angiogenesis on HUVECs. The impact of miR210 inhibitor and GLGZD on tube formation (a) (scale bar = 500 μm), NO content and activity of eNOS (b), relative protein expression of VEGF and VEGFR2 (c, d) in HUVECs after OGD/R for each group. Data are presented as mean ± SD. *p < 0.05, **p < 0.01 vs. control, ^#^*p* < 0.05, ^##^*p* < 0.01 vs. OGD/R, ^Δ^*p* < 0.05, ^ΔΔ^*p* < 0.01 vs. miR210 inhibitor.

To further confirm the regulation of miR-210 on the angiogenesis process via HIF1α, transfection of cells with miR-210 inhibitor *in vitro* was conducted. As expected, transfection of cells with miR-210 inhibitor robustly decreased miR-210 level (Figure 5(a), *p* < 0.01). Tube formation of HUVECs results indicated that miR-210 inhibitor impeded GLGZD-mediated facilitating effects on cell viability and tube formation in HUVECs (Figure 6(a), *p* < 0.01). Next, angiogenesis-related markers were examined, the protein levels of NO, eNOS, VEGF and VEGFR were obviously repressed by miR-210 inhibitor (Figure 6(b–d), *p* < 0.01).

### miR210 inhibitor attenuates the GLGZD-mediated neuroprotective effects after I/R injury

**Figure 7.**
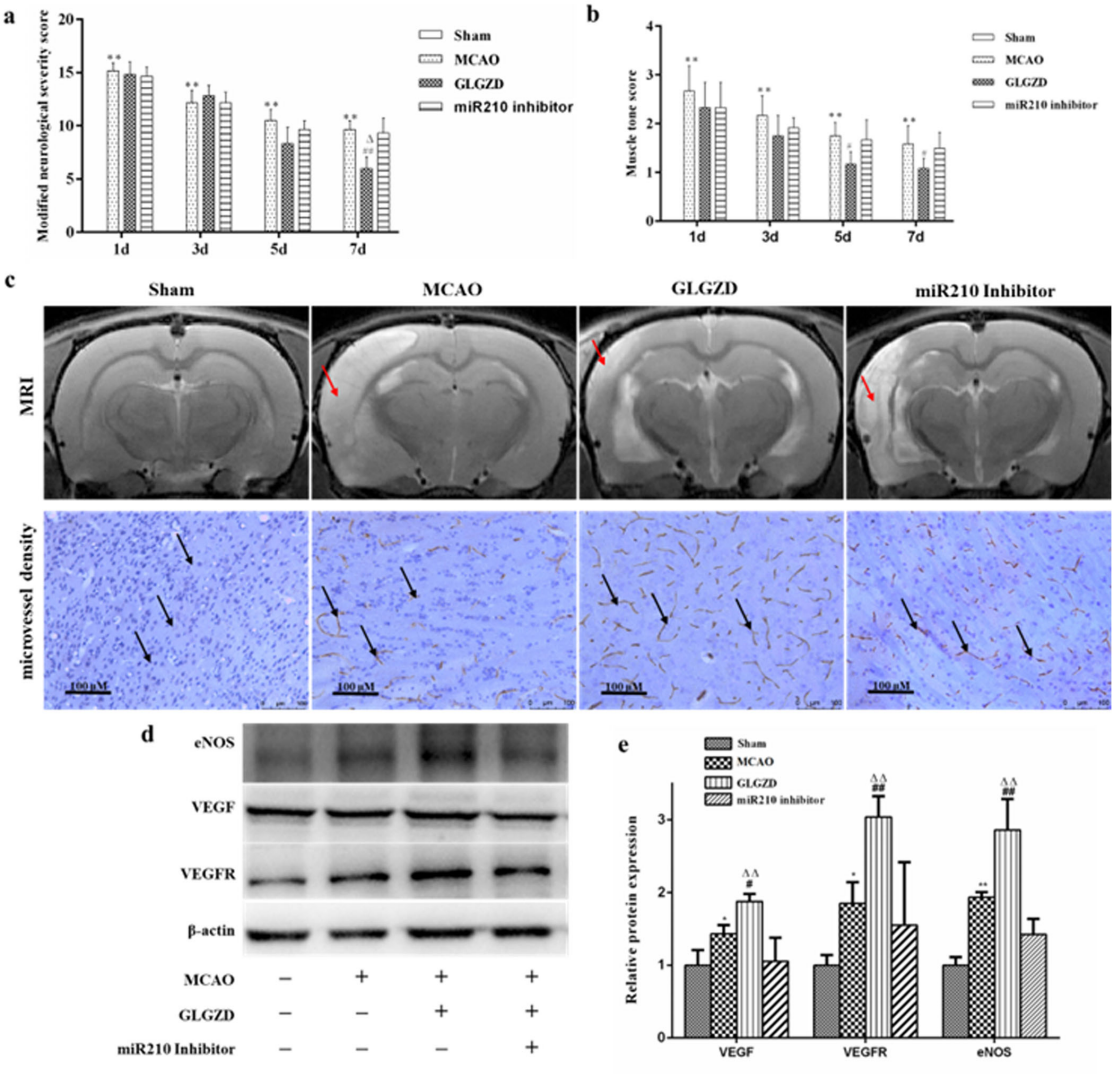
miR210 inhibitor attenuates the GLGZD-mediated neuroprotective effects after I/R injury. The impact of miR210 inhibitor and GLGZD on neurologic deficit (a, b), MRI and infarct volume and HE stain (c) and representative photographs of immunohistochemistry staining of CD34 (indicated by black arrows), relative protein expression of eNOS, VEGF and VEGFR2 (d, e) in rats for each group. Data are presented as mean ± SD. **p < 0.01 vs. sham, ^#^*p* < 0.05, ^##^*p* < 0.01 vs. MCAO, ^Δ^*p* < 0.05, ^ΔΔ^*p* < 0.01 vs. miR210 inhibitor.

To further explore the role of miR-210 on the neuroprotective effects induced by GLGZD under cerebral ischemia, neurologic deficit, area of cerebral infarction, histological and morphological assessment were evaluated in rats. The results showed that the functional recovery induced by GLGZD was obviously diminished by miR210 inhibitor (Figure 7(a,b)), as well as area of cerebral infarction, histological and morphological changes (Figure 7(c)). Next, we tested the influence of miR210 inhibitor on the GLGZD induced increase of microvessel density and regulation of VEGF, eNOS and NO. It was found that administration of miR210 inhibitor dramatically suppressed the induction of microvessel density, VEGF, eNOs and NO caused by GLGZD (Figure 7(c–e)). These results demonstrated that miR210 deficiency dampened the neuroprotective effects of GLGZD.

### miR-210 directly targeted HIF1α

**Figure 8.**
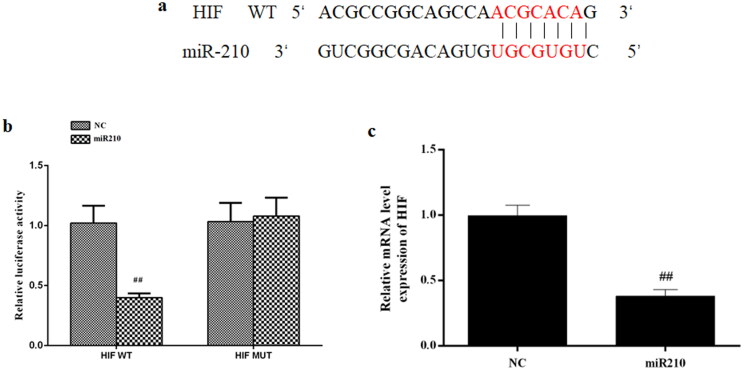
HIF is the direct target of miR-210. (a) Predicted binding sites between miR-210 and HIF. (b) Relative luciferase activities of WT-HIF 3′UTR and MUT-HIF 3′UTR in cells transfected with mimics NC or miR210 mimics. (c) Effect of miR210 mimics on HIF mRNA expression. Data are presented as mean ± SD. ^##^*p* < 0.01 vs. NC.

It has been indicated that miR-210 positively regulates HIF1α expression, implying that HIF1α might be a downstream target of miR-210. To test that, we first performed bioinformatic analysis by TargetScan and found some complementary binding sites between miR-210 and HIF1α mRNA (Figure 8(a)). Then the dual luciferase assay was used to directly validate this interaction, the result showed that miR-210 mimics greatly increased the luciferase activities of WT-HIF1α 3′UTR but not MUT-HIF1α 3′UTR wherein the predicted binding sites were mutated (Figure 8(b)).

## Discussion

Ischemic stroke is usually caused by cerebral vascular occlusion, which leads to local cerebral ischemia and hypoxia, resulting in corresponding neurological dysfunction. Accumulating evidence has shown that angiogenesis in cerebral ischemia/reperfusion injury is beneficial for neurovascular remodelling as it improves cerebral blood flow and metabolism (Beck and Plate 2009). It has been reported that angiogenesis is positively correlated with survival and recovery in patients following ischemic stroke (Krupinski et al. 1994). Nevertheless, physiological post-stroke angiogenesis is very limited, therapeutic enhancement of angiogenesis appears promising to yield potential therapeutic strategy for ischemic stroke. Fortunately, GLGZD, a famous classic clinical decoction applied for several years, has prominent neuroprotective effect on the treatment of ischemic stroke. However, its mechanism remains less clear.

This study confirms that GLGZD significantly improves functional recovery after cerebral ischemia. Simultaneously, GLGZD could increase microvessel density following cerebral ischemic injury, exhibited effective angiogenesis. Angiogenesis further provides nutritive blood flow and favourable microenvironment for the protection of neurons. As expected, the results of MRI, HE and Nissl’s staining showed that GLGZD significantly alleviated the brain damage and reduced neuronal apoptosis. The high expression of CD34 in GLGZD group indicates the pro-angiogenic effects. All of the results of our study reconfirm the beneficial effects of angiogenesis on functional outcomes, as per the previous reports (Chen et al. 2007; Wang et al. 2014).

The pro-angiogenic effects may relate to the regulation of GLGZD on pro-angiogenic factors such as VEGF, VEGFR, NO and eNOS. It is well known that VEGF is a critical factor involved in the process of neoangiogenesis. The binding of VEGF and VEGFR2 activates multiple angiogenic signals, which coordinate with each other to form functional vessels (Greenberg and Jin 2013). *In vivo* and *in vitro* studies have revealed that GLGZD notably up-regulated the pro-angiogenic factors VEGF, VEGFR, NO and eNOS. NO and eNOS play an important role in promoting endothelial cell proliferation, endothelial cell migration and tube formation (Gentile et al. 2013). *In vitro* data have shown that GLGZD MS administered to HUVECs caused the cells proliferation, migration and tube formation. In a second study, Wang et al. (2005) found that VEGF protected neurons from cell death directly rather than only by advancing angiogenesis. It is noteworthy that angiogenesis work for functional recovery after cerebral ischemia is a complex procedure and is regulated by multiple factors. Thus, further study is needed to unveil the underlying mechanisms.

More important, cerebral hypoxia induces stress response leading to HIF activation, which, in turn, stimulates the release of VEGF. Then, the binding of VEGF to VEGFR-2 expressed on endothelial cells, triggers many angiogenesis factors production, including eNOS, NO, EGF and PAI-1. Increasing evidence has shown that the HIF/VEGF signalling pathway plays a primary role in stroke induced angiogenesis (Beck and Plate 2009). In this study, the expression changes of HIF-1α spontaneously increased after MCAO and OGD by immunostaining and Western blotting assay, and was further up-regulated by GLGZD, in parallel with angiogenesis factors, as VEGF, VEGFR, NO and eNOS. In addition, it was found that GLGZD could up-regulate the expression of PLCγ and PKC. As we know, PKC can inhibit PHD2 activation, which in turn inhibits the pervasiveness of HIF degradation leading to an increased angiogenesis. The study also found that blocking of PKC *in vivo* results in inhibition of VEGF-induced angiogenesis.

As reported, miRNAs, such as miR210, regulating endothelial cell survival, differentiation and migration, play a crucial role in angiogenesis (Ren et al. 2016; Bavelloni et al. 2017). In the present study, it was found that miR210 was increased significantly after GLGZD treatment in HUVECs exposed to OGD, and the miR210 inhibitor completely abolishes the beneficial effects of GLGZD on cell migration, tube formation and cell survival *in vitro*. Moreover, the modulation of proangiogenic factors by GLGZD was also reversed by miR210 inhibitor. Our results were in line with a previous study which showed that miR-210 is involved in angiogenesis after cerebral ischemia, and is correlated closely with VEGF expression (Jiang et al. 2020; Rahmati et al. 2021). Furthermore, our data have demonstrated that the miR210 was increased significantly after GLGZD treatment *in vivo*, and down-regulation of miR210 abolished the beneficial effects of GLGZD on microvessel density and functional recovery.

Notably, through preliminary bioinformatic analysis, we identified that HIF may be a downstream target of miR210. miR210 inhibitor suppressed the increased expression of HIF following OGD. In addition to that, dual luciferase reporter analysis further confirms that HIF was the direct target of miR210. These data suggested that the interaction of miR210 and HIF greatly contributes to neoangiogenesis.

In combination of both *in vivo* and *in vitro* results, it is demonstrated that GLGZD enhanced angiogenesis via activating the miR210/HIF/VEGF pathway.

## Conclusions

GLGZD exerted a neuroprotective effect by enhanced angiogenesis via activation of the miRNA210/HIF/VEGF signalling pathway *in vivo* and *in vitro*. Our findings would be useful for the clinical practice of GLGZD, as an effective angiogenic formula for stroke recovery.
